# Zooming in and out: a holistic framework for research on maternal, late foetal and newborn survival and health

**DOI:** 10.1093/heapol/czab148

**Published:** 2021-12-09

**Authors:** Neha S Singh, Andrea K Blanchard, Hannah Blencowe, Adam D Koon, Ties Boerma, Sudha Sharma, Oona M R Campbell

**Affiliations:** London School of Hygiene and Tropical Medicine, Keppel Street, London WC1E 7HT, UK; Department of Community Health Sciences, University of Manitoba, R070-771 McDermot Avenue, Winnipeg, MB R3E 0T6, Canada; London School of Hygiene and Tropical Medicine, Keppel Street, London WC1E 7HT, UK; Department of International Health, Johns Hopkins Bloomberg School of Public Health, 615 North Wolfe Street, Baltimore, MD 21205, USA; Department of Community Health Sciences, University of Manitoba, R070-771 McDermot Avenue, Winnipeg, MB R3E 0T6, Canada; CIWEC Hospital and Travel Medical Center, G.P.O. Box 12895, Kapurdhara Marg, Kathmandu 44600, Nepal; London School of Hygiene and Tropical Medicine, Keppel Street, London WC1E 7HT, UK

**Keywords:** Maternal health, newborn health, foetal health, stillbirths, mixed methods, low- and middle-income countries, conceptual framework

## Abstract

Research is needed to understand why some countries succeed in greater improvements in maternal, late foetal and newborn health (MNH) and reducing mortality than others. Pathways towards these health outcomes operate at many levels, making it difficult to understand which factors contribute most to these health improvements. Conceptual frameworks provide a cognitive means of rendering order to these factors and how they interrelate to positively influence MNH. We developed a conceptual framework by integrating theories and frameworks from different disciplines to encapsulate the range of factors that explain reductions in maternal, late foetal and neonatal mortality and improvements in health. We developed our framework iteratively, combining our interdisciplinary research team’s knowledge, experience and review of the literature. We present a framework that includes health policy and system levers (or intentional actions that policy-makers can implement) to improve MNH; service delivery and coverage of interventions across the continuum of care; and epidemiological and behavioural risk factors. The framework also considers the role of context in influencing for whom and where health and non-health efforts have the most impact, to recognize ‘the causes of the causes’ at play at the individual/household, community, national and transnational levels. Our framework holistically reflects the range of interrelated factors influencing improved MNH and survival. The framework lends itself to studying how different factors work together to influence these outcomes using an array of methods. Such research should inform future efforts to improve MNH and survival in different contexts. By re-orienting research in this way, we hope to equip policy-makers and practitioners alike with the insight necessary to make the world a safer and fairer place for mothers and their babies.

**Key messages**

- Integrated research on maternal, late foetal and newborn health (MNH) is lacking, and conceptual frameworks for MNH research are usually topic- and/or discipline-specific.
- This article presents a novel and holistic conceptual framework for MNH research reflecting a range of interrelated factors leading to improved MNH and survival.
- The framework aims to re-orient maternal and newborn health research and in turn equip policy-makers and practitioners alike with the insight necessary to improve maternal, late foetal and newborn outcomes.

## Introduction

Over the past few decades, many countries have achieved notable declines in maternal and neonatal mortality (Collaboration, 2018). However, there are still many preventable deaths, hence the continued inclusion of these outcomes in the Sustainable Development Goal (SDG) targets (Boerma *et al.*, 2018; Braveman and Gruskin, 2003). Late foetal mortality (stillbirth) rates are not an explicit SDG target (Qureshi *et al.*, 2015) and are still widely neglected; yet, they share many of the same biomedical and social causes as maternal and neonatal mortality. Preventing all these deaths and improving health are amenable to multiple preventive and curative interventions as well as a range of programmatic approaches to ensure these interventions are adopted (Bhutta *et al.*, 2008).

Research to understand the reasons for countries’ success in improving maternal, late foetal and newborn health (MNH) and reducing mortality will provide valuable insights for others with similar aims to do so appropriately and comprehensively. Factors affecting MNH are complex and operate at many levels, so it can be difficult to eludicate which were necessary conditions for the successes observed. There has been a proliferation of health policies, programmes and specific interventions to directly or indirectly improve these outcomes, such as improving access to quality obstetric services or care for sick and small newborns, hygiene and infection management, and more broadly improving women’s nutrition and encouraging early and exclusive breastfeeding. Still, much remains unknown about the relative contribution and interrelated impact of such interventions and how they are affected by socio-demographic, economic, cultural, environmental and epidemiological shifts in different contexts or by the organization of health and other relevant services (Boerma *et al.*, 2018; Braveman and Gruskin, 2003).

Conceptual frameworks are central to this process of discovery because they provide a cognitive means of rendering order to the world around us. In public health, researchers integrate theories and evidence into conceptual frameworks to display the relationships among a range of constructs or variables, often in relation to health outcomes (Miles and Huberman, 1994). They are less propositional than theoretical frameworks and allow researchers to integrate theories or concepts in new ways and apply them to guide research. As concepts and their interrelationships are better understood through research and practice, such frameworks are ideally refined based on new evidence (Mosley and Chen, 1984; McCarthy and Maine, 1992; Marsh *et al.*, 2002; Kramer *et al.*, 2019; George *et al.*, 2018). Frameworks related to maternal and newborn or child health to date have taken different approaches and vary in whether they concentrate on ‘zoomed-in’, selective interventions or broader ‘zoomed-out’ approaches. Some focus on proximate drivers such as biomedical determinants or risk factors (Mosley and Chen, 1984; McCarthy and Maine, 1992). Others consider intermediate factors such as programme and service delivery outputs, as well as effective coverage of interventions across the continuum of care (Tanahashi, 1978; Raven *et al.*, 2012; Campbell *et al.*, 2016; Amouzou *et al.*, 2019). Yet others focus on more distal factors such as the roles of socioeconomic contexts (Sabot *et al.*, 2018; Rosenfield, 1985; Croghan *et al.*, 2006; George *et al.*, 2015) and health policy implementation and health system inputs in directly, or indirectly, influencing the health of women and their children (Shiffman and Smith, 2007; Sheikh *et al.*, 2011; Qiu *et al.*, 2018; George *et al.*, 2019). However, few have conceptualized the factors influencing reductions in neonatal mortality and stillbirths, or explicitly integrated them with maternal mortality, despite the close interlinkage of their causes and related interventions (Costello and Osrin, 2005; Marsh *et al.*, 2002). For example, an estimated 80% of all newborn deaths result from three preventable and treatable conditions—complications due to prematurity, intrapartum-related deaths (including birth asphyxia) and neonatal infections—which in part reflects a suboptimal intrauterine environment or poor maternal health (Blencowe *et al.*, 2013; World Health Organization, 2012).

We developed a conceptual framework by integrating theories and frameworks from different disciplines to encapsulate the range of factors that explain reductions in MNH. This framework was developed in the context of the Exemplars in MNH study to orient seven mixed-methods case studies in low- and middle-income countries (LMICs)—Bangladesh, Ethiopia, India, Morocco, Nepal, Niger and Senegal—with better than expected progress in reducing maternal and neonatal mortality since 2000, where we aim to learn lessons that can further advance efforts and inform strategies in other settings (Exemplars, 2021). While our focus is on the range of factors explaining mortality reductions, we anticipate that the framework’s utility extends beyond this to guide other researchers seeking to explain or explore specific or multiple factors in relation to improving MNH in a flexible manner. Furthermore, rather than seeing each component of the framework separately as ‘determining’ the outcomes, the framework helps to remind us to consider how various factors worked together over time, in a given context.

## Methods

We developed the framework iteratively, combining the results of a critical review of the literature with the knowledge and experience from our interdisciplinary research team and other global experts (Grant and Booth, 2009). Our research team members and technical advisory group of global experts were diverse in terms of disciplinary expertise (maternal and/or newborn health; social sciences; biostatistics; epidemiology; health economics; health policy and systems research; medical anthropology), affiliations (academic institutions, civil society organizations, governmental actors and non-governmental organizations) and countries (Senegal, Morocco, Niger, Ethiopia, India, Nepal, Bangladesh, South Africa, Brazil, Canada, United Kingdom and USA).

To start this process, we purposively searched and gathered peer-reviewed and grey literature from 1960 onward for evidence, theories and frameworks that had been used to understand the factors influencing MNH, and particularly the reduction of maternal and neonatal mortality and stillbirths (Novak and Cañas, 2006; Crawford, 2019). Supplementary Annex 1 shows all the factors and domains that we initially considered in MNH. The research team also sourced additional relevant literature iteratively during the process of developing the framework.

**Figure 1.**
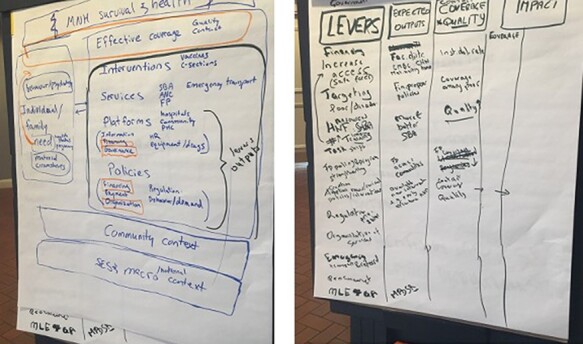
Outputs of workshop group work to display the draft framework components

The co-authors who were involved since the inception of the Exemplars MNH study met in a workshop in January 2020 to review and discuss the domains and factors identified in the previous step and to brainstorm in groups which components were needed and how they related to the others. In two groups, we narrowed down the key components and drafted visual frameworks to display how they were interrelated. Next, the groups presented and discussed their drafts (Figure 1) and reached consensus on the most relevant approach to studying the factors influencing MNH and survival.

After the workshop, we used virtual meetings to develop the first draft of the framework with all Exemplars in MNH co-authors and shared this with a technical advisory group of multidisciplinary global health experts for validation and then incorporated their inputs. Finally, we defined the framework’s sub-components and related indicators as a team (see Supplementary Annex 2) based on the relevant literature, the co-authors’ knowledge, and finalized the framework presented in this paper.

## Results

### Theoretical underpinnings of the framework

We categorized 53 conceptual frameworks found in our critical review into the following broad areas: (1) frameworks on factors influencing maternal and/or newborn and late foetal (stillbirth) mortality and related health impacts; (2) frameworks on the continuum of care in relation to effective coverage and health service delivery; (3) health policy and system research (HPSR) frameworks relating to MNH and (4) frameworks focusing on contextual factors related to MNH.

### Frameworks on factors influencing maternal, late foetal and newborn health and survival

Several conceptual frameworks have focused on the proximate and intermediate factors specifically influencing maternal and/or neonatal/infant/child mortality and stillbirths and their related causes. The concept of the mother–child dyad has been emphasized at least since the 1996 WHO ‘Mother-Baby Package’ (World Health Organization, 1996), but has required renewed focus in the past decade to ensure programmes jointly support mothers and babies and prevent stillbirths (Kinney *et al.*, 2016; Chou *et al.*, 2015). For example, this would mean that, ‘any effort to train midwives in care during childbirth must include essential interventions for the newborn baby; maternal death audits must also investigate newborn deaths; and postnatal home visits by community health workers must assess the mother’s as well as the newborn baby’s health and needs’ (Starrs, 2014). This emphasis was reiterated in two series of articles in *The Lancet* on maternal health in 2006 and 2016. Similarly, the maternal and newborn health community developed joint objectives for preventing maternal mortality, neonatal mortality and stillbirths, including to strengthen care around the time of childbirth when most of these deaths occur (Chou *et al.*, 2015).

Mosley and Chen (1984) and McCarthy and Maine (1992) introduced influential frameworks on child and maternal mortality, respectively. These focused on individual-level or household-level and some intermediate-level (care provision) determinants, which influenced mother’s and children’s survival. Many of the components identified are relevant to, but did not explicitly include, neonatal mortality or stillbirths. For example, these include maternal age, parity and birth interval, environmental contamination and nutrient deficiency that affects the baby’s survival (Mosley and Chen, 1984), as well as the direct causes of maternal mortality including the range of interrelated complications or indirect causes that relate to women’s health status (McCarthy and Maine, 1992).

A paper by Thaddeus and Maine (1994) recognized that most direct and indirect causes of maternal death could be prevented with timely medical treatment, and conceptualized three delays of deciding to seek care, identifying and reaching the facilities, and receiving appropriate and adequate treatment, and how these were affected by socio-economic/cultural factors, service accessibility and quality of care (Thaddeus and Maine, 1994). This framework was expanded by Gabrysch and Campbell in 2009, based on an evidence-based review of the household/individual perceived need, as well as community and societal factors leading to utilization of maternity health services for both normal and complicated births. More recently, Kramer *et al.* (2019) assessed community-level determinants for equity in maternal survival in the USA, focusing on social, behavioural, transportation, reproductive, and general health environments at individual, community and societal levels, and how these together affect maternal health status and risk of mortality (Kramer *et al.*, 2019).

Some frameworks also bring in a secular element and signal the concept of ‘transitions’, in terms of linking mortality levels and related socio-demographic context, with implications for the required interventions. In particular, the ‘obstetric transition’ framework posits important socio-demographic and health system factors that may differ at different stages or levels of maternal mortality (Souza *et al.*, 2013; Chaves *et al.*, 2015). At stages with the highest maternal mortality levels, it indicates that most deaths are from direct causes or from communicable diseases like malaria. As mortality declines, and with increasing access and quality of skilled childbirth care, indirect causes become more important, and eventually, most deaths are due to chronic-degenerative disorders (Souza *et al.*, 2013; Chaves *et al.*, 2015).

There were no analogous frameworks for transitions in levels of stillbirths or neonatal mortality. We found one source on the epidemiological transition towards declining mortality and increasing risk of over-medicalizing maternal, perinatal and newborn health, coupled with a neglect of addressing broader factors through community health interventions (Costello and Osrin, 2005). One framework for newborn health in LMICs more broadly emphasizes the balance between preventive care (19 routine behaviours) and curative care (14 special behaviours) and is rooted at the community level (Marsh *et al.*, 2002). The Pathway to Survival model designed to support the Integrated Management of Childhood Illness approach, has enriched the data gathered on care seeking for child illnesses and supported the development of demand- and supply-side interventions, and its related Pathway Analysis social autopsy format has been updated to improve the assessment of neonatal deaths in addition to child deaths (Kalter *et al.*, 2011).

### Frameworks on health service delivery and intervention coverage

Several frameworks relevant to assessing influences on MNH have focused on linking the proximate and intermediate factors: how health impact is achieved by bringing together frameworks on the continuum of reproductive, maternal, newborn, child and adolescent health and nutrition (RMNCAH + N) interventions with those on improving equitable and effective coverage, service delivery and programme platforms. As specific targets in Millennium Development Goals 4 and 5 [World Health Organization (WHO)], maternal and child health was situated within an expanding continuum of care that encompassed a broad set of evidence-based interventions needed to effectively improve health outcomes for women, children and adolescent girls. Newborn health and reduction of stillbirths have also been included in these frameworks during the SDG era. These RMNCAH + N interventions were conceived across a temporal continuum of care, from preconception to postnatal care for MNH, and a spatial continuum of care, involving linkages between community, outreach and facility-based services (Kerber *et al.*, 2007).

Relating to the spatial continuum, there has been a large emphasis on community-based RMNCAH + N interventions to improve MNH and survival, in combination with facility-based service delivery (Rosato *et al.*, 2008). Programmes have used a mix of community mobilization and health promotion approaches through group meetings and/or home visits by community health workers (Rosato *et al.*, 2008), with growing evidence on the effects of these efforts to improve both overall perinatal and newborn health outcomes and close equity gaps between socio-economic groups (Schiffman *et al.*, 2010; Schleiff *et al.*, 2017; Blanchard *et al.*, 2019). Renewed focus on primary health care has supported efforts to link health with other aspects of social well-being and development over the long term to achieve multisectoral action, moving towards integrating ‘health in all policies’ (Kuruvilla *et al.*, 2018).

There is a recognized need to better understand the processes by which and contexts in which community approaches can best enhance maternal, late foetal and neonatal mortality reduction (Gram *et al.*, 2019) and to explore when community approaches are inappropriate. For example, there is an issue with the implicit definition of the level of care defined as ‘primary care’, as too often primary care is conflated with the lowest level of the health system (e.g. care delivered via community health workers). However, ‘primary care’ for childbirth should take place at minimum in a health centre, if not a hospital, because of the specific challenges of predicting risk and the efficiency needed to address complications for mothers and newborns through skilled or specialist care and equipment, which is therefore inappropriate at the lowest level health facilities (Campbell *et al.*, 2016).

Turning to the delivery of RMNCAH services and related interventions, Tanahashi’s (1978) framework on ‘effective coverage’ first depicted coverage as the number of people contacting services (such as for antenatal care, skilled birth attendance or postnatal care), those receiving interventions (like tetanus toxoid, iron folic acid tablets and so on; Tanahashi, 1978) and expanding on the WHO’s framing of coverage by including availability, accessibility, quality and acceptability (World Health Organization, 2016). Since then, frameworks have refocused and expanded on the original concept of ‘effective coverage’. These recognize the need not only to increase populations’ contact with health services and interventions through improved availability, accessibility and acceptability, but also emphasize that they need sufficient readiness and quality to have an impact on health and survival (Amouzou *et al.*, 2019; Larson *et al.*, 2017; Carvajal–Aguirre *et al.*, 2017; Boerma *et al.*, 2018; Marsh *et al.*, 2020).

Conceptualization of effective coverage includes quality of care dimensions on which the MNH literature has expanded. The WHO’s definition of quality care emphasizes that services be effective, safe, timely, equitable, integrated and people-centred (WHO). These quality components also require respectful, equitable and integrated services as described earlier in relation to coverage and equity across the RMNCAH continuum of care. This definition of quality of care is consistent with more recent definitions that emphasize both the technical and experiential dimensions of quality. The 2018 Lancet Commission on high-quality health systems in the SDG era emphasizes both processes of care (including competent and respectful care and systems, and positive user experiences) and quality impacts (i.e. health impacts, trust in the system and economic benefits; Kruk *et al.*, 2018). Raven *et al.*’s (2012) review on quality in MNH care defines it in Donabedian’s terms as structure (health policy and system inputs), process (service delivery) and resulting outputs and outcomes (Raven *et al.*, 2012). In *The Lancet’s* 2016 Maternal Health series, Koblinsky *et al.* (2016) advocate for the following priority actions to improve quality of maternal health care: (1) prioritize quality maternal health services that respond to the local specificities of need and meet emerging challenges; (2) promote equity through universal coverage of quality maternal health services, including for the most vulnerable women; (3) increase the resilience and strength of health systems by optimizing the health workforce and improve facility capability; (4) guarantee sustainable finances for maternal-perinatal health; (5) and accelerate progress through evidence, advocacy and accountability (Koblinsky *et al.*, 2016). Similar priority actions are required to improve newborn health care.

### Health policy and systems research frameworks

HPSR has become increasingly recognized as an important multidisciplinary approach, with relevance for understanding how to optimize policies and health systems that improve the delivery of services and interventions that impact MNH and survival (Gilson, 2012; Sheikh *et al.*, 2014; Walt *et al.*, 2008). Related to policy prioritization, Shiffman’s novel comparative analyses shed new light on factors influencing national policy agendas for addressing maternal mortality, including transnational influence (norm promotion and resource provision), domestic advocacy (political community cohesion, political entrepreneurship, credible indicators, focusing events and clear policy alternatives) and national political environment (political transitions and competing health priorities; Shiffman and Garcés del Valle, 2006; Shiffman and Smith, 2007).

Reflecting the need for better integration of policy and health systems, Sheikh *et al.* (2011) characterized three key lenses that reflect changing emphases in HPSR: functional, complexity and socio-political lenses. An analysis of these historical shifts in political contexts traced the functional lens back to the shift away from comprehensive primary health care in the 1970s towards decentralization in healthcare organization and a growing number of actors (including private sector) in the 1980s. This led to a focus beyond just health service delivery and administration, towards understanding how policy was translated into functional or ‘technical’ components or ‘hardware’ needed to strengthen health systems (van Olmen *et al.*, 2012). Frameworks in this vein that continue to inform current concepts of ‘hardware’ include the World Bank ‘control knobs’, such as governance, financing and demand issues (Weber *et al.*, 2010; Shakarishvili *et al.*, 2010) and the WHO’s ‘six health system building blocks’ comprising of service delivery, health workforce, health information, techno-medical products, financing, leadership and governance (World Health Organization, 2006; 2010).

This was followed by an appreciation not only of the health systems’ functional focus on hardware but also its complexity and how these interrelated with the ‘software’, including power, relationships, ideas, interests, norms, values and ultimately the role of people that shape health policy and systems (Sheikh *et al.*, 2011). There has also been more emphasis on the socio-political contexts and particularly social construction of policy-making and health systems’ software and hardware, which influence each other within socio-political spheres (van Olmen *et al.*, 2012; Sheikh *et al.*, 2011). More recently, the HPSR field has moved to working more substantially on scaling up, sustainability, political priority and resilience (Qiu *et al.*, 2018).

George *et al.* outlined the HPSR lenses and levels in a framework to understand the drivers of governance for RMNCAH. They argued that attention is still paid predominantly to the hardware, but less to the social relationships and health systems dynamics at the micro-, meso- and macro-levels that affect outcomes (George *et al.*, 2019). A joint analysis by the WHO working groups Every Newborn Action Plan and Ending Preventable Maternal Mortality also related HPSR lenses and levels to addressing maternal and newborn mortality and stillbirths by including an objective to strengthen both the hardware and software of health systems, as well as engaging families and communities, and improving the use of data for decision-making and accountability (Chou *et al.*, 2015).

### Context-focused frameworks for MNH

Of critical importance is to account not only for intentional policies and programmes designed to target health outcomes, but also to recognize the contextual processes at play in each setting over time. Although most frameworks discussed above include some elements of context in relation to MNH, health system inputs or service delivery, few focus more explicitly on contextual influences on MNH. Sabot *et al.* propose contextual factors across various domains—epidemiological, demographic, health service provision, health system, economics, infrastructure, education and environment—as the broader milieu influencing MNH programme implementation (Sabot *et al.*, 2018). In their framework, context is categorized as ‘structural’, meaning that it is changing slowly and mainly at the macro level, or ‘situational’, meaning it is changing relatively quickly and more likely to affect MNH outcomes, including socio-demographic and fertility characteristics or sanitation (as well as health service quality and coverage and health system hardware, which are covered earlier as intentional actions to improve MNH).

Context was also intentionally explored in the Good Health at Low Cost study in their analyses of how health systems optimized cost-efficient strategies to tackle maternal, neonatal and child mortality (Balabanova *et al.*, 2013). Balabanova et al.’s more recent study was informed by the original Good Health at Low Cost work in the 1980s (Rosenfield, 1985) and Croghan *et al.*’s (2006) research that shed light on the roles of social, economic and political contexts in improving health in four low-income countries. Those case studies found that beyond health policy and systems changes, key contextual factors contributing to maternal and child health included good governance and political commitment to accountability and action; resilient, effective and flexible bureaucracies and institutions; and improvements in infrastructure, gender equity, female empowerment and education in line with the Social Determinants of Health framework (Balabanova *et al.*, 2013).

More recently, George *et al.* developed a conceptual framework that delineates contextual factors into four overlapping spheres (community, health facilities, health administration and society) with cross-cutting issues (awareness, trust, benefits, resources, legal mandates, capacity-building, the role of political parties, non-governmental organizations, markets, media, social movements and inequalities). Their review of contextual factors highlights the dynamic relationships and broader structural elements that facilitate and/or hinder the role of health committees, which are critical to mediating between communities and health services in many health systems (George *et al.*, 2015).

Several frameworks report gender as a cross-cutting contextual issue that affects pregnancy and childbirth and impacts women’s and newborn’s health on many levels (Bill and Melinda Gates Foundation, 2020). Notably, Morgan *et al.*’s framework for studying gender in health systems summarizes gender power relations as being constituted by norms, perceptions, ideologies, and beliefs (i.e. how values are defined), roles, time allocation, and division of labour (i.e. who does what), access to resources (i.e. who has what), and rules or decision-making (i.e. who decides) (Morgan *et al.*, 2016). These domains can be examined at the household and individual levels in terms of interpersonal relationships and decision-making, but also how they interact with social norms and structures at the community and macro-level contexts (Morgan *et al.*, 2016).

### Introducing a holistic conceptual framework for research on MNH

**Figure 2.**
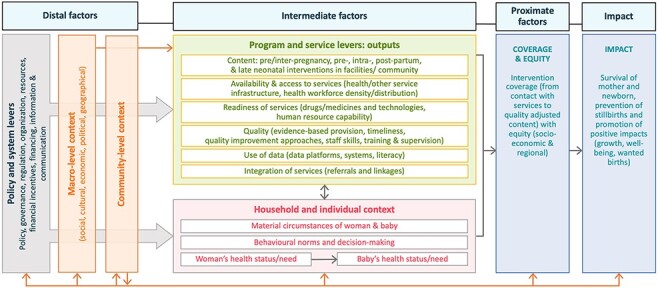
Holistic conceptual framework for maternal, late foetal and newborn survival and health

The conceptual framework presented in Figure 2 offers an interdisciplinary, integrated approach to understanding the drivers of improvements in MNH and survival. It was developed by integrating literature on past evidence-based frameworks, with expert knowledge and experience from different settings and disciplines.

Figure 2 depicts how distal-, intermediate- and proximate-level factors may affect the health and survival of women and babies. First, we outline distal factors that influence MNH outcomes, i.e. health policy and system levers and macro-level contextual factors. On the far left, we draw attention to the multisectoral policy and system levers, which are tools used by governments to improve MNH specifically, as well as decisions that are not taken with a focus on MNH, but may have an enormous impact on MNH (e.g. efforts to improve girls’ education gender inequity or infrastructure in underserved parts of the country). Government actions include those to develop or change policies, to increase financial and human resources for MNH programmes or related health services, to regulate and monitor the public and private sector and to organize services in different ways. Macro- and community-level contextual factors (e.g. social, cultural, economic, political, infrastructural or environmental) importantly modulate the effects of governments’ changes in the health policy and system levers on programme and service outputs for MNH. This may include the accountability and responsiveness of the health system and services to local government and community structures.

Next, we outline intermediate-level factors influencing MNH outcomes. Health policy and system levers at the distal level aim to specifically influence health programme and health service outputs, i.e. more concrete outputs of government actions at the intermediate level. These comprise (1) programme content, i.e. pre-/inter-pregnancy, pre-/intra-partum and postnatal contacts at both health facility and community levels; (2) access to health services, including location and infrastructure for health and other services, health workforce density and distribution, and financial support; (3) readiness of health services, including availability of essential drugs, medicines, equipment and technologies; (4) quality of health services, including competent care and positive experiences; (5) integration of services, including timely referrals and linkages between different levels of the health system (e.g. community, primary health-care structures, secondary and tertiary care facilities); and (6) health information use for decision-making for improved patient care. The programme and service improvements are critical intermediate steps towards increasing intervention coverage and equity and ultimately impacting MNH. Macro-level contextual factors can also directly influence the intermediate programme and service outputs, which in turn affect levels and equity in coverage of key MNH interventions. These levers are also interlinked with household- and individual-level contexts at the intermediate level, including material circumstances (such as household assets and income), behavioural norms and decision-making, and health status or need of the women and babies concerned.

The coverage of interventions adopted by families, across the continuum of MNH care—promotive, preventive and curative—are included in the proximate factors in the framework. Intervention coverage is more directly associated with maternal and newborn survival and other health impacts compared to more distal or intermediate factors. In this framework, we recognize that moving from contact (e.g. use of ANC) to quality-adjusted coverage of specific interventions (Amouzou *et al.*, 2019) is a key factor affecting MNH outcomes, and that these interventions must be equitably delivered between socio-economic groups and geographical regions, both to reduce mortality overall (since deaths cluster in certain more marginalized groups) and for principles of justice and equity.

Altogether, these distal, intermediate and proximate factors and the levers used to influence them have an impact on the outcomes at the far right of the framework, namely maternal, late foetal and newborn mortality and morbidity across key time periods, i.e. pre-/inter-pregnancy, and pre-/intra-partum and postnatal, and over time. Specifically, it is possible to use the framework to consider the reasons for changes in both the levels, patterns and biomedical causes of maternal, late foetal and neonatal mortality in a given setting. Cause of death patterns change substantially as mortality levels change. The lack of reliable cause of death information in most LMICs is however disconcerting. Estimates and changes in cause-specific maternal and newborn mortality differ considerably between studies and have been hard to track consistently (Graham *et al.*, 2016). Timing of death may serve as a proxy for causes of death. For example, a meta-analysis of neonatal mortality studies in South-east Asia and sub-Saharan Africa showed the predominance of preterm births and intrapartum causes in the first days and first week, while infectious diseases have greater impact after the first week (Sankar *et al.*, 2016). Meanwhile, at higher levels of stillbirth rate (>25 per 1000 births), ∼50% are due to antepartum causes and 50% due to intra-partum causes. As stillbirth rates decline, the proportion of intra-partum goes down (Lawn *et al.*, 2011). The timing of maternal death (antepartum, during delivery and postpartum) is also associated with specific causes. For example, haemorrhage, often a lead cause of maternal death at higher levels of mortality, occurs predominantly in the postpartum period (Black *et al.*, 2016).

## Discussion

Our critical review of relevant frameworks and evidence informed the different sections of our framework. Our framework explicitly drew on those conceptualized by McCarthy and Maine (1992) for maternal mortality, Mosley and Chen (1984) on proximate determinants of under-five and infant mortality, as well as others outlined above on intermediate and distal factors. It also drew on the concept of transitions to understand the patterns in the main causes, contexts and solutions at different levels of mortality. The framework relates the outcomes to intentional efforts within the health sector. This included the proximate factors on coverage and equity of interventions that specifically relate to past frameworks on the evidence-based interventions encompassed by the spatial and temporal dimensions of the RMNCAH + N continuum of care needed to improve mortality and health among mothers and babies. Moving to the intermediate factors within the health sector, we drew on the aforementioned frameworks conceptualizing service delivery and programme platforms for RMNCAH + N services and interventions.

To identify the levers that were intentional efforts to influence health service and intervention coverage at the intermediate level, the framework draws largely on the health policy and systems implementation features from the World Bank control knobs (Weber *et al.*, 2010), the under-five mortality- and stunting-focused Exemplars study frameworks (Gates Ventures, 2021), the WHO’s health systems building blocks (World Health Organization, 2010), George *et al.*’s (2019) lenses and levels framework, and the Countdown to 2015 country case-study frameworks for health policy and service research in relation to RMNCAH + N (Singh *et al.*, 2016). We also considered models of multisectoral action that aim also to improve MNH (Kuruvilla *et al.*, 2018). Across these levels of the framework, we drew from frameworks that generally or specifically included factors that relate to other sectors or unintentional contextual factors. We organized them as factors relating to the individual (woman and baby) and household at the intermediate levels and the community and macro-level at distal levels, which may variably influence the health policy and system inputs, programme and services outputs, the coverage and equity of interventions, as well as survival. Supplementary Annex 2 defines the framework’s components and related indicators that can be used to map the framework in a given context.

### Opportunities and challenges for applying the framework

Our objective for applying the framework to guide our mixed-methods case studies in seven exemplar countries was to study how intentional actions (agency) and contextual factors (structure) together have contributed to greater than expected reductions in mortality. To do this, we developed an iterative analytical approach to allow each country case study to tailor the framework using mixed methods that are conducted concurrently but integrated at multiple stages (Greene *et al.*, 1989; Fetters *et al.*, 2013).

Our multi-country research is in progress, but the aim is to narrow down the broad set of potential drivers for deeper investigation by first broadly mapping the contextual and health policy and system changes that could have shaped the MNH outcomes in each setting through the review of documents and literature. Concurrently, quantitative survey analyses will describe the trends in maternal and neonatal mortality and stillbirths, and coverage and equity of RMNCAH interventions, which will guide specific hypotheses on which drivers have contributed. We aim to use qualitative review of databases and documents of health service or programme outputs to identify connections between the most important health service and programme drivers and the MNH outcomes (Greene *et al.*, 1989). Quantitative analyses will statistically describe changes in these health service and programme factors where data are available. LiST analyses on the contribution of RMNCAH + N interventions to mortality reduction will also point to the significant socio-demographic, epidemiological, macro-economic and/or health system factors to study using further analyses. These analyses are intended to refine hypotheses on the most relevant health system inputs, as well as contextual factors, to study further using qualitative and quantitative data.

At the explanatory stage, we will seek to study the relative importance and nature of the key drivers’ contributions to improved MNH outcomes (Greene *et al.*, 1989). Quantitatively, multivariate analyses will help to understand how changes in the composition of the population may affect maternal and neonatal mortality rates when data permit and the relative contribution of the identified drivers to the changes in MNH outcomes. Meanwhile, qualitative in-depth interviews with purposively selected key informants will help to study how policy and programme development and implementation processes led to improved MNH coverage and outcomes and the role of contextual factors. Finally, a synthesis of results across study contexts or regions will be valuable to compare the mixed-methods results and seek to explain divergent findings. This will also provide an opportunity to further refine and adapt the framework components and how they link together to impact MNH.

Given the complex nature of the research to understand drivers of MNH improvement, there are challenges that we may anticipate in operationalizing the framework. One may be the availability of data and integration of methods with different assumptions about causality. Given the breadth of topics, studies applying the framework may face challenges in maintaining depth or complexity. Finally, there may be challenges for tracing the processes that connect the framework’s components, and particularly looking at changes over time. There may be limited availability of data or recall of past events. This may relate particularly to the implementation processes or ‘software’ components, in part because they are rarely intentionally documented. We hope that focusing research on what has worked well to improve MNH through a mixed-methods approach may help to illuminate the aspects that glue the framework components together (Morgan, 2007).

To address these potential challenges, the framework may be most applicable to interdisciplinary teams of researchers and practitioners with varying backgrounds, expertise and experience that work together to understand the factors relating to maternal and newborn health and survival that are of interest in their contexts. While our case study approach draws on integrated mixed methods to consider the potential range of factors related to MNH and survival to analyse within different country contexts, others could readily draw on this framework in empirical research to explore or explain their dimensions of interest using a range of methods such as scoping reviews, qualitative case studies and various quantitative analyses.

## Conclusions

Our framework is the first to holistically reflect the range and contextual nature of the interrelated factors leading to improved MNH and survival. To develop this framework, we integrated available evidence and conceptual components—including health policy and systems levers or intentional actions that governments and policy-makers can implement to improve MNH; health service delivery and coverage of interventions across the continuum of care, and the role of epidemiological and behavioural risk factors, at different levels of mortality. It also considers the role of context in influencing for whom and where health and non-health efforts have the most impact, to recognize ‘the causes of the causes’ at play at the individual/household, community, national and transnational levels (Sabot *et al.*, 2018). The framework lends itself to studying how different factors work together to influence the outcomes using an array of methods. Such research should inform future efforts to improve maternal and newborn health and survival in different contexts. By re-orienting research in this way, we hope it will equip policy-makers and practitioners alike with the insight necessary to make the world a safer and fairer place for mothers and their babies.

## Supplementary Material

czab148_Supp

## Data Availability

The data underlying this article are available in the article and in its online supplementary material.
