# Supplementary material for: Zooming in and out: a holistic framework for research on maternal, late foetal and newborn survival and health
Source: Health Policy Plan. 2021 Dec 9;37(5):565–74. doi: 10.1093/heapol/czab148 (PMC9113153; doi:10.1093/heapol/czab148)
Supplement: czab148_Supp [file czab148_supp.zip › 2021.12.15 Exemplars MNH conceptual framework paper_web annexes.docx]

# Web Annexes

**Annex 1.** Detailed list of factors and domains that may influence maternal and newborn health


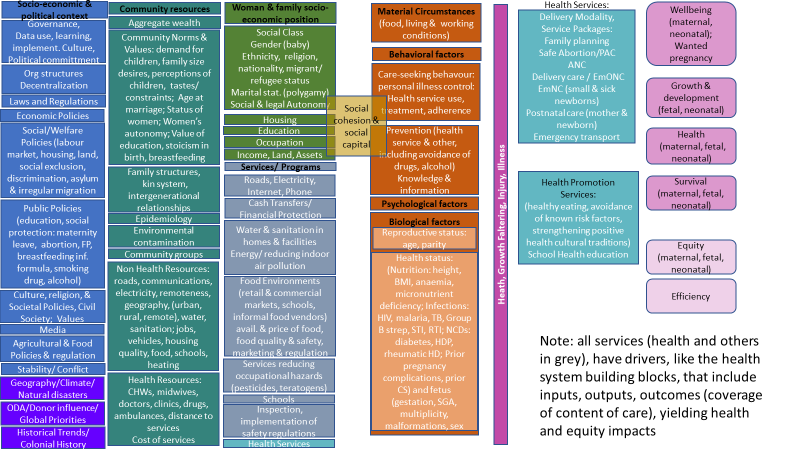


**Annex 2.** Indicators, data sources and methods for each framework component

Generic approaches to analysing the relationships between levers, coverage and health status impacts, and the role of contextual factors, are shown in Tables 1-5. The tables present a menu of indicators, data sources and quantitative and/or qualitative methods that may be used to study each component in the framework (to be adapted based on data quality and availability and using the hypotheses and literature relevant in each setting). These components include:

- distal health policy and system levers (Table 1);
- distal contextual factors at the macro- and community-levels (Table 2);
- intermediate household context and individual epidemiologic and behavioural factors (Table 3);
- intermediate program and service levers (Table 4);
- proximate MNH intervention coverage, and health impacts (Table 5).

Table 1: Indicators, data and methods for distal-level policy and system levers

| **Indicators** | **Data sources** | **Methods** | |
| --- | --- | --- | --- |
|  |  | **Quantitative** | **Qualitative** |
| **Health policy and system levers** | | | |
| Policies | | | |
| Policy formulation related to health generally:   - Human right to health - Universal access to health care and services - Integration of MNH in national health strategy - National MNH strategies and implementation plans - MNH institutional arrangements - Equity-oriented policies - Standards of quality MNH care and referrals - Health worker deployment/ retention policies   Policy formulation specific to:   - Reproductive health (SRH services, family planning, safe abortion, and commodities) - Maternal health (antenatal, intrapartum, emergency obstetric (EmOC), and postnatal care, & maternal death reviews) - Newborn health (essential newborn care and resuscitation, management of preterm births and Kangaroo Mother Care, postnatal care and exclusive breastfeeding, care for small and sick newborns, perinatal death reviews) - Stillbirths | - WHO MNCAH policy database - UNFPA data bases on sexual and reproductive health policies - Documents and reports on national health strategy and plans, annual health reports - Qualitative information from policy makers |  | - Health policy analysis with desk review and key informant data to understand the MNH-relevant policies, their scope, resource allocations, and timelines |
| Governance & leadership | | | |
| - National and subnational leadership for MNH including level of engagement, political connections, visibility - Costed national implementation plan(s) for RMNCH available - National coordinating body that looks at RMNCH or its components - National human rights institution authorised to consider matters related to RMNCH - Maternal and perinatal deaths reviews - Civil society involvement in review of national maternal, newborn and child health programs - Parliamentary engagement in MNH - Accountability, including regular transparent inclusive reviews - Degree of managerial autonomy; decentralization | - Guidelines and reports, electronic resources - Qualitative key informant interviews or group discussions with leaders in national/ subnational public, civil society or private sector |  | - Health system analyses of document review and qualitative key informants’ perspectives on leadership, power and relationships, and ‘will’ for implementing policies, and delivering RMNCH services and programs |
| Resources and organization of services | | | |
| Health resources:   - Training/deployment of health workers, especially cadres critical for MNH - Service delivery strategies, e.g., community health workers - Supply systems for essential medicines and commodities   Organization of services and supply chains:   - Organization of administrative units for health care provision (integration and referral systems) - Organization of (sub)national health management for MNH programs including non-health professionals and community health workers - Organization of district health management teams for MNH programs - Organization of MoH in relation to antenatal, childbirth, postnatal and family planning and abortion services | - Guidelines and reports, electronic resources | - Training institutions and intake/ outputs - Analysis of distribution of essential medicines from a wholesaler to health service facilities (sub-national) - Analysis of health system strength (subnational) | - Health system analysis of document review |
| Financing | | | |
| Financial inputs for MNH / RMNCH:   - Federal and regional governments - Donors - Out-of-pocket (OOP) expenditures and financial protection measures - Public and private insurance: systems and quality of services | - Financial data from national and subnational health accounts (NHA, PER) - WHO Global Health Expenditure database - Research on OOP expenditures & financial protection - Qualitative informant interviews with financing experts and program staff | - Quantitative analysis of online databases - Analysis of (sub) national budgets and finance allocation and OOP expenditures - Analysis of utilization of public and private insurance | - Qualitative analysis of resource allocation for health service outputs and specifically MNH programs |
| Payment | | | |
| - Payment-for-performance and incentives to providers, including doctors, nurses, public health managers, midwives, community health workers, and other relevant cadres | - NHA and (sub) national budgets, financial reports - Qualitative informant interviews with health care providers | - Quantitative analysis of payments scheduled/ made; comparisons of areas with and without interventions (or performance analysis at subnational level) | - Qualitative analysis of key informants’ attitudes and experiences with payment structures |
| Regulation | | | |
| - Formal standards for safe and ethical practices in health care services and program implementation - Regulation of the private sector: presence of country database of private sector service providers / facilities; accreditation system for private providers; reporting requirements for private providers - Systems to monitor availability and quality of services (public, private, non-governmental) | - Legal or other regulatory documents for (sub)national health organizations and providers - Qualitative key informant interviews/group discussions with program implementers |  | - Document review and qualitative analysis of perspectives on regulations or monitoring systems and their effectiveness |
| Information | | | |
| - Regular system of accountability: monitoring (timely and accurate), review of progress and performance (inclusive, transparent), remedial action (translation into implementation) - Plans with clear indicators and targets for MNH that are monitored - Presence and use of health information databases, including HMIS and program monitoring & evaluation at different levels of the health system | - Electronic national, regional and program-specific databases - Qualitative key informant interviews/ group discussions with health management teams/program analysts | - Quantitative analysis of quality of HMIS or facility data | - Document review and qualitative analysis on program analysts’ use of data for decision making, performance review and improvement |
| Communication | | | |
| - Tools and platforms for communicating messages for evidence-informed MNH practices to households and individuals (public, civil society and private sectors) | - Electronic resources, program documents - Qualitative informant interviews with program implementers |  | - Document review, qualitative analysis of the use of tools and platforms |

Table 2: Indicators, data and methods for macro- and community-level contextual factors

| **Indicators** | **Data sources** | **Methods** | |  |
| --- | --- | --- | --- | --- |
|  |  | **Quantitative** | **Qualitative** |  |
| **Macro-level context** | | | |  |
| Transnational context | | | |  |
| - Prioritization by international organizations - Diffusion of ideas across borders/region - Effectiveness of transnational advocates; e.g., CAARMA - Donor resources available/ DA/Donor influence | - Aggregated indicators/scores from national databases and statistical compendia, e.g., The Creditor Reporting System (CRS) | - Trends over time, comparison to other countries | - Document review; - Qualitative syntheses |  |
| Geography/climate/natural disasters | | | | |
| - Distances; remoteness, - Urban-rural residence - Population size, density & growth rate - Climate - History of natural disasters and their effects | | - Aggregated indicators/scores from national databases and statistical compendia, e.g., Demographic Yearbook (UN); UN Department of Economic and Social Affairs; World Pop | - Descriptive |  |
| Past colonial rule/historical trends | | | |  |
| - Tradition of health cadres, clinical education |  |  | - Descriptive analyses |  |
| - Political context | | | |  |
| - National political stability; leadership strength - Form of governance/structure of government and political parties - Strength of civil society - Ideological orientation of regime - State penetration of society - Governance and corruption - Changes in government through violent and non-violent means/ conflict - Ethnic diversity and fragmentation (more socio-cultural); social integration & cohesion - Participation in political process - Universal franchise and political engagement - Decentralization - Community involvement | - Aggregated indicators/scores from national databases and statistical compendia, e.g., Demographic Yearbook (UN); UN Department of Economic and Social Affairs; World Bank Country reports - Transparency international |  | - Descriptive analyses |  |
| Social and cultural context | | | |  |
| - Organization and practice of religions - Societal values (perceptions of value of the newborn child can impact on both care-seeking practices & whether health systems invest in newborn-specific components. Personhood of young neonates may be delayes so illness/death viewed in a fatalist manner - Media | - Aggregated indicators/scores from national databases and statistical compendia, e.g., World Values Survey International Social Survey Programme | - Trends over time, comparison to other countries | - Document review; Qualitative syntheses |  |
| Macroeconomic policies & context | | | |  |
| - Strength of national economy - Per capita GDP; GNP - Income inequality; income/asset distribution - Strength of transport and communications infrastructure - Resources available to health sector - Foreign aid from bilateral & international development partners - Management and integration of aid with existing international resources | - Aggregated indicators/scores from national databases and statistical compendia of financial data from national accounts, - World Bank documents online databases - ODA/OECD documents - National Health Accounts | - Trends over time, comparison to other countries | - Document review; Qualitative syntheses |  |
| Public/social/welfare policies, laws & regulations | | | |  |
| - Education levels and policies (basic & secondary); literacy levels - Social protection: maternity leave - Equity oriented services (education, health, food/nutrition - Urban-rural equality - Social welfare orientation to development   - Land reform   - Educational programs   - Support for basic necessities   - Preventive orientation - Labour market, housing - Approach to social exclusion, discrimination, asylum and irregular migration | - Aggregated indicators/scores from national databases and statistical compendia including MDG/SDG tracking; Global Development Finance (World Bank); - Human Development Report and statistical database (UN), Social Security Worldwide (ISSA), ILOSTAT (ILO), - OECD iLibrary, - UN Data and contributing sources e.g., World Health Organization Statistics WHO Regional Health Observatory Data - Repository, World Bank open data; World Development Indicators; UN Gender Statistics, - Global Health Data Exchange (GHDx), Global Health Workforce statistics - State of the World’s Midwifery - Humanitarian data exchange (HDX) | - Trends over time, comparison to other countries | - Document review; Qualitative syntheses |  |
| Agricultural/ food policies & regulation | | | |  |
|  | - Aggregated indicators/scores from national databases and statistical compendia including FAO | - Trends over time, comparison to other countries | - Document review; Qualitative syntheses |  |
| Intersectoral linkages for health | | | |  |
| - Mechanisms for linkages for health - Incentives for linkages - Recognition that health is socially determined |  | - Trends over time, comparison to other countries | - Document review; Qualitative syntheses |  |
| **Community-level context** | | | |  |
| Community non-health resources (can also be considered nationally) | | | |  |
| - Geography of community (urban, rural, remote), - Altitude for malaria; temperature - Roads, vehicles, transport - Communications (phone ownership/internet, incl. in facilities) - Electricity - Water & sanitation in homes & facilities - Indoor air pollution - Housing quality - Schools - Community-level aggregated wealth levels (average wealth quintile, or human development type indices) - Cash transfers/ financial protection - Jobs, employment - Food environments (retail & commercial markets, schools, informal food vendors) avail. & price of food, food quality & safety, marketing & regulation - Community groups/political organizations - Inspection, implementation of safety regulations - Services reducing occupational hazards (pesticides, teratogens) | - DHS, MICS, other national household surveys e.g., LSMS - Aggregated indicators/scores from subnational databases and statistical compendia - Geographic databases | - Descriptive and multilevel quantitative analysis | - Document review; Qualitative syntheses |  |
| Community norms & values | | | |  |
| - Fertility: demand for children, family size desires, perceptions of children, tastes/ constraints - Age at marriage - Status of women - Women’s autonomy - Value of education - Ethnicity/religion (cultural & religious traditions) - Stoicism in birth/norms around childbirth - Breastfeeding | - DHS, MICS, other national, and subnational household surveys - Aggregated sub-national indicators/scores from databases & statistical compendia - Literature including Ethnographic compendia, e.g., eHRAF World Cultures; World Values Survey International Social Survey Programme | - Descriptive and multilevel quantitative analysis | - Document review; Description of context |  |
| Family structures | | | |  |
| - Kinship systems - Intergenerational relationships - Spousal separation (labor migration) | - Ethnographic compendia e.g., eHRAF World Cultures - DHS, MICS, other national household surveys for spousal separation | - Descriptive quantitative analysis | - Document review; Description of context |  |

Table 3: Indicators, data and methods for household & individual contextual factors

| **Indicators** | **Data sources** | **Methods** | |
| --- | --- | --- | --- |
|  |  | **Quantitative** | **Qualitative** |
| **Household & individual context** | | | |
| Health status/need | | | |
| Reproductive status:   - Maternal parity distribution (prevalence of high parity) - Maternal age distribution (prevalence of older age at birth; could also do younger) - Previous caesarean section   Health status:   - Maternal anemia - Maternal short stature - Maternal underweight/ obesity - HIV prevalence rate in women of reproductive age - Low-birth weight prevalence - Prevalence of twins (multiplicity) - Sex of child (unlikely to be important unless imbalanced) - Previous stillbirth (rate) - Unwanted pregnancy - Consanguinity levels - Micronutrient deficiency/folic acid supplementation - Infections, malaria, TB, Group B strep, STIs (syphilis prevalence), RTI; - NCDs: diabetes, HDP, rheumatic HD - Malformations | - DHS/MICS - Aggregated indicators - Literature including aggregated indicators/scores from databases and statistical compendia | - Levels and trends analysis; - Inequality analysis by subnational areas and socio-economic position - Multivariate analysis of neonatal mortality; decomposition analysis - Association with impacts, coverage, program and policy timelines; interrupted time series | - Description of burden of ill-health and whether services are targeted to these problems |
| Household/woman/baby’s material circumstances | | | |
| - Living/housing conditions, incl. indoor air pollution - Social class - Ethnicity, religion, nationality, migrant/ refugee status - Marital status (polygamy) - Social & legal autonomy - Education and literacy - Occupation, working conditions - Income, land, assets - Gender/sex of baby - Food (diversity & security); optimal micronutrients for foetus | - DHS/MICS and other surveys including nutrition/food security - Literature including aggregated indicators/scores from databases and statistical compendia and ethnographic | - Levels and trends analysis; - Equity analysis by subnational areas and socio-economic position - Multivariate analysis of neonatal mortality - Association with coverage, program and policy timelines | - Description of context and whether services are targeted/ responsive to these issues |
| Household/woman’s behavioral norms and decision making | | | |
| Behaviours:   - Personal illness control; health service use and care seeking - Treatment, adherence, prevention - Harmful behaviours: incl. drugs, alcohol, smoking - Knowledge & information   Psychology:   - Social cohesion & social capital | - DHS/MICS and other surveys incl. on smoking, drug and alcohol - Literature including aggregated indicators/scores from databases and statistical compendia and sociologic literature | - Levels and trends analysis | - Description of context |

Table 4: Indicators, data and methods for intermediate-level program and service levers

| **Indicators** | **Data sources** | **Methods** | |
| --- | --- | --- | --- |
|  |  | **Quantitative** | **Qualitative** |
| **Program and service levers** | | | |
| Program contents and service delivery strategies | | | |
| - Implementation plans and processes of interventions (facility- and community-based) for pre/inter-pregnancy, antenatal, Intrapartum/birth, and postpartum (postnatal) periods - Levels of services where deliveries take place/ (health centre/hospital; EmONC capability) and type of provider (doctor, midwife), policies and training - Levels and providers of other services & national policy/ trainings for newborn care   - Primary care     - Essential newborn care, cadres in the community and training for detection of small and/or sick newborns, both immediately after home birth and throughout neonatal period     - recognition of small and/or sick newborn etc. (plus referral)   - Level 2 –– Newborn facilities (and number with any respiratory support e.g. CPAP)   - Level 3 – full NICU   - Policies and services for newborns post discharge (or for home births coming from community):   - IMNCI – ‘newborn’ included in IMCI, Community Case Management (CCM) of newborn - Sector of care (private/public) | - Program implementation plans, guidelines, reports, evaluations - Qualitative key informant interviews with program managers and implementers |  | - Document review, qualitative analysis of key informants’ experiences and perceptions of program planning and implementation processes |
| Access to services including MNH services | | | |
| - Infrastructure for health and other services (social and economic) - Health workforce density and distribution (e.g. midwives, nurses, neonatal nurses, doctors, clinical officers with specific training, obsetritcians , neonatologists, pediatricians) - Provision of beds (numbers and density) (e.g. inpatient newborn cots with CPAP/ respiratory support cots) - Financial protection (compensation or user fee removal, cash transfers) - Utilization of public/private health insurance | - Data on facilities (numbers and density), health workforce, and beneficiary compensation or user fee removal | - Descriptive and multilevel quantitative analysis; analysis of linked Health Facility Census and census/DHS data | - Document review on barriers to accessing care |
| Readiness of services | | | |
| - Appropriate drugs and medicines (stockouts and supply chains) - Up-to-date technologies, supplies and equipment for family planning, abortion care, ANC, PNC, routine delivery care, EmONC, inpatient care for small and sick newborns and treatment of sepsis | - Data on the distributions, stockouts and supply chains for drugs and medicines, technologies, supplies, equipment - ETAT (neonatal part) | - Descriptive and multilevel quantitative analysis |  |
| Quality of services | | | |
| - Information use - Human resources for health (training, supportive supervision and skills) - Length of stay, pre-discharge check, weighing baby at birth for facility births - Experience of care (as a driver of demand) | - Facility data (HMIS or DHIS, SPA, SARA), program data and reports - Observations of care - Qualitative key informant interviews with health providers, supervisors, and service users | - Descriptive and multilevel quantitative analysis | - Qualitative analysis of facility observations and key informants’ perspectives on the quality of MNH care provided and received |
| Use of data |  |  |  |
| - Data platforms - Health information systems or other service monitoring tools - Health information literacy | - Data system records | - Analyses on facility data quality | - Reports or published literature on data systems quality and use for informing programmes and services |
| Integration of services | | | |
| - Referral mechanisms during pregnancy, labour and for sick newborns; Identification of at-risk pregnancies and referral to appropriate level of care for delivery - Vehicles and ambulances - Linkages between care providers | - Data on referral mechanisms, ambulatory services, and linkages between primary, secondary and tertiary providers (private & public) - Qualitative key informant interviews/group discussions | - Descriptive quantitative analysis of types and numbers of referral pathways and linkages - Facility preparedness for referral (transport & communication) | - Qualitative analysis of key informants’ experience of referrals and their efficiency |

Table 5: Indicators, data and methods for proximate determinants of intervention coverage and health impacts

| **Indicators** | **Data sources** | **Methods** | |
| --- | --- | --- | --- |
|  |  | **Quantitative** | **Qualitative** |
| **Intervention coverage (Source: Countdown; Global Strategy; EPMM and ENAP core indicators)** | | | |
| **Maternal**   - Demand for family planning services satisfied - Comprehensive abortion care and post-abortion care - Antenatal care (4+ and 1+); timing of antenatal visits (1st trimester), care with skilled provider - Antenatal quality components: blood pressure, blood and urine sample taken, and iron supplementation - ART coverage for pregnant women with HIV - IPT & bed nets for pregnant women (in settings w/malaria) - Skilled birth attendance - Institutional delivery - Postnatal care for mothers (within 48 hours) - C-section deliveries, by wealth quintile & Robson classif. - Iron/folic acid supplementation during pregnancy - Uterotonic immediately after birth   **Newborn**   - Neonatal tetanus protection - Postnatal care for babies - Early initiation of breastfeeding - Proportion of newborns reported weighed at birth - Thermal care: drying and delayed bath for 24 hours - Clean cord care - Exclusive breastfeeding for 0-5 months (for first month) - Newborn resuscitation - Kangaroo mother care, feeding support - Treatment of neonatal sepsis | - DHS, MICS, other national, and subnational household surveys; - HMIS data; Health facility surveys linked to household surveys; program specific data | - Levels and trends and disparities by equity stratifiers (subnational regions, place of residence, wealth quintiles, maternal education, maternal age, sex of child) - Multivariate analysis of factors that contributed to changes in coverage indicators that showed dramatic changes - Association with program/service levers and health impacts |  |
| **Impacts** | | | |
| - Maternal mortality ratio, maternal mortality rate, lifetime risk of maternal death - Neonatal mortality rate (early and late, by sex) - Stillbirth rate and perinatal mortality rate | - DHS/MICS; HMIS; country-specific surveys | - Levels and trends analysis, and equity analyses by subnational areas, maternal age, residence and socioeconomic characteristics - Trends in causes of maternal and newborn deaths and cause-specific analyses - Lives Saved Tool Analysis, multivariate analysis of neonatal mortality, decomposition analysis - Association with coverage and policy and program timeline |  |
